# 4-Hy­droxy­anilinium perchlorate dihydrate

**DOI:** 10.1107/S1600536810025365

**Published:** 2010-07-03

**Authors:** Xue-qun Fu

**Affiliations:** a Ordered Matter Science Research Center, Southeast University, Nanjing 210096, People’s Republic of China

## Abstract

In the crystal structure of the title compound, C_6_H_8_NO^+^·ClO_4_^−^·2H_2_O, inter­molecular N—H⋯O and O—H⋯O hydrogen bonds occur. The protonated amine cations and the perchlorate anions are linked through the water mol­ecules, and the hy­droxy groups of the cations and the anions are linked through the water mol­ecules. The cations are connected to the perchlorate anions *via* inter­molecular N—H⋯O hydrogen bonds. In addition, the crystal structure exhibits weak inter­molecular C—H⋯π inter­actions.

## Related literature

For background to phase transition materials, see: Li *et al.* (2008 ▶); Zhang *et al.* (2009 ▶)
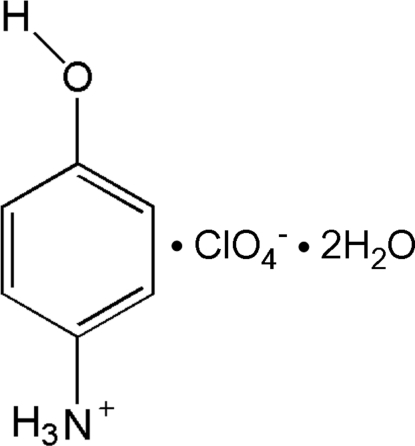


## Experimental

### 

#### Crystal data

C_6_H_8_NO^+^·ClO_4_^−^·2H_2_O
*M* *_r_* = 245.62
Orthorhombic, 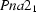

*a* = 24.341 (5) Å
*b* = 5.253 (1) Å
*c* = 7.824 (2) Å
*V* = 1000.4 (4) Å^3^
*Z* = 4
Mo *K*α radiation
μ = 0.40 mm^−1^
*T* = 298 K
0.40 × 0.30 × 0.20 mm

#### Data collection

Rigaku SCXmini diffractometer
Absorption correction: multi-scan (*CrystalClear*; Rigaku, 2005 ▶) *T*_min_ = 0.866, *T*_max_ = 0.923
9517 measured reflections
2275 independent reflections
1986 reflections with *I* > 2σ(*I*)
*R*_int_ = 0.052

#### Refinement

*R*[*F* ^2^ > 2σ(*F* ^2^)] = 0.048
*wR*(*F* ^2^) = 0.097
*S* = 1.11
2275 reflections
168 parameters
8 restraints
H atoms treated by a mixture of independent and constrained refinement
Δρ_max_ = 0.21 e Å^−3^
Δρ_min_ = −0.53 e Å^−3^
Absolute structure: Flack (1983 ▶), 1049 Friedel pairs
Flack parameter: 0.00 (7)

### 

Data collection: *CrystalClear* (Rigaku, 2005 ▶); cell refinement: *CrystalClear*; data reduction: *CrystalClear*; program(s) used to solve structure: *SHELXS97* (Sheldrick, 2008 ▶); program(s) used to refine structure: *SHELXL97* (Sheldrick, 2008 ▶); molecular graphics: *SHELXTL* (Sheldrick, 2008 ▶); software used to prepare material for publication: *SHELXTL*.

## Supplementary Material

Crystal structure: contains datablocks I, global. DOI: 10.1107/S1600536810025365/lx2151sup1.cif

Structure factors: contains datablocks I. DOI: 10.1107/S1600536810025365/lx2151Isup2.hkl

Additional supplementary materials:  crystallographic information; 3D view; checkCIF report

## Figures and Tables

**Table 1:** Hydrogen-bond geometry (Å, °) *Cg*1 is the centroid of the C1–C6 benzene ring.

*D*—H⋯*A*	*D*—H	H⋯*A*	*D*⋯*A*	*D*—H⋯*A*
O1—H1*O*⋯O2*W*^i^	0.75 (3)	2.10 (3)	2.801 (3)	156 (4)
N1—H1*N*⋯O4	0.79 (5)	2.34 (5)	3.016 (4)	144 (4)
N1—H2*N*⋯O1*W*^ii^	0.98 (4)	1.98 (5)	2.951 (4)	168 (4)
N1—H3*N*⋯O1*W*	1.00 (5)	1.97 (5)	2.972 (4)	175 (4)
O1*W*—H1*AW*⋯O3^iii^	0.79 (5)	2.40 (7)	3.089 (3)	146 (8)
O1*W*—H1*BW*⋯O5	0.83 (4)	2.47 (6)	3.083 (4)	132 (5)
O2*W*—H2*AW*⋯O4	0.93 (4)	2.28 (4)	3.068 (4)	143 (4)
O2*W*—H2*BW*⋯O1^iv^	0.77 (3)	2.17 (3)	2.937 (3)	173 (5)
C2—H2⋯*Cg*1^iv^	0.93	2.88	3.677 (3)	144

Symmetry codes: (i) 
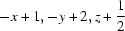
; (ii) 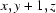; (iii) 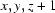; (iv) 
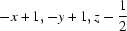
.

**Fig. 1.**
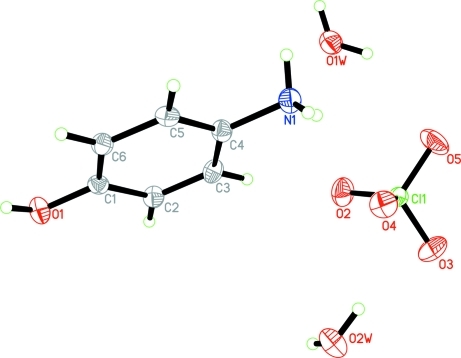
The molecular structure of the title compound with the atom numbering scheme. Displacement ellipsoids are drawn at the 30% probability level. H atoms are presented as a small cycles of arbitrary radius.

**Fig. 2.**
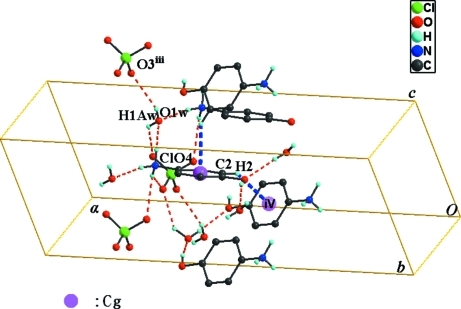
N—H···O, O—H···O and C—H···π interactions (dotted lines) in the crystal structure of the title compound. Cg denotes the ring centroid. [Symmetry codes: (i) - x + 1, - y + 2, z + 1/2; (ii) x, y + 1, z; (iii) x, y, z + 1; (iv) - x + 1, - y + 1, z - 1/2.]

## supplementary crystallographic information

### Comment

As a continuation of our study of phase transition materials, including organic ligands (Li *et al.*, 2008), metal-organic coordination compounds (Zhang *et al.*, 2009), organic–inorganic hybrids, we studied the dielectric properties of the title compound, unfortunately, there was no distinct anomaly observed from 93 K to 350 K, suggesting that this compound should be not a real ferroelectrics or there may be no distinct phase transition occurred within the measured temperature range. Here, we report the crystal structure of the title compound (Fig. 1).

The asymmetric unit of the title compound is made up of a 4–hydroxyanilinium cation cation wherein the non-hydrogen atoms are practically co-planar with a mean deviation of 0.015 (2) Å, a perchlorate anion and two solvent molecules of water (Fig. 1). The crystal packing (Fig. 2) is stabilized by intermolecular N—H···O , O—H···O hydrogen bonds and weak intermolecular C—H···π interactions. (Table 1). Both the protonated amine cations and the perchlorate anions are linked through the water molecules, and the hydroxy groups of the cations and the anions are linked through the water molecules. Additionally, the cations are connected to the perchlorate anions via intermolecular N—H···O hydrogen bonds.

### Experimental

1.09g (10 mmol) 4–aminophenol was firstly dissolved in 10ml ethanol, to which perchloric acid aqueous solution (70% *w*/*w*) was then added under stirring until the PH of the solution was ca. 6. Ethanol was added until the precipitated substrates disappeared. Colorless prism single crystal for X–ray was obtained by  the acid solution slow evaporated at room temperature after two days.

### Refinement

Aryl  H atoms were positioned geometrically and refined using a riding model, with C—H = 0.93 Å.  *U*_iso_(H) = 1.2*U*_eq_(C). The other H atoms attached to N and O atoms were found difference maps using restraints for O—H bond distances (O—H = 0.85 (5) Å) and H—O—H angles (H···H =1.35 (10) Å). Their displacement parameters were freely refined.

### Crystal data

**Table:**

C_6_H_8_NO^+^·ClO_4_^−^·2H_2_O	*F*(000) = 512
*M**_r_* = 245.62	*D*_x_ = 1.631 Mg m^−^^3^
Orthorhombic, *P**n**a*2_1_	Mo *K*α radiation, λ = 0.71073 Å
Hall symbol: P 2c -2n	Cell parameters from 4523 reflections
*a* = 24.341 (5) Å	θ = 3.1–55.2°
*b* = 5.253 (1) Å	µ = 0.40 mm^−^^1^
*c* = 7.824 (2) Å	*T* = 298 K
*V* = 1000.4 (4) Å^3^	Prism, colourless
*Z* = 4	0.40 × 0.30 × 0.20 mm

### Data collection

**Table:**

Rigaku SCXmini diffractometer	2275 independent reflections
Radiation source: fine-focus sealed tube	1986 reflections with *I* > 2σ(*I*)
graphite	*R*_int_ = 0.052
Detector resolution: 13.6612 pixels mm^-1^	θ_max_ = 27.5°, θ_min_ = 3.1°
ω scans	*h* = −31→31
Absorption correction: multi-scan (*CrystalClear*; Rigaku, 2005)	*k* = −6→6
*T*_min_ = 0.866, *T*_max_ = 0.923	*l* = −10→10
9517 measured reflections	

### Refinement

**Table:**

Refinement on *F*^2^	Secondary atom site location: difference Fourier map
Least-squares matrix: full	Hydrogen site location: difference Fourier map
*R*[*F*^2^ > 2σ(*F*^2^)] = 0.048	H atoms treated by a mixture of independent and constrained refinement
*wR*(*F*^2^) = 0.097	*w* = 1/[σ^2^(*F*_o_^2^) + (0.0423*P*)^2^] where *P* = (*F*_o_^2^ + 2*F*_c_^2^)/3
*S* = 1.11	(Δ/σ)_max_ = 0.001
2275 reflections	Δρ_max_ = 0.21 e Å^−^^3^
168 parameters	Δρ_min_ = −0.53 e Å^−^^3^
8 restraints	Absolute structure: Flack (1983), 1049 Friedel pairs
Primary atom site location: structure-invariant direct methods	Flack parameter: 0.00 (7)

### Special details

**Table:**

Geometry. All esds (except the esd in the dihedral angle between two l.s. planes) are estimated using the full covariance matrix. The cell esds are taken into account individually in the estimation of esds in distances, angles and torsion angles; correlations between esds in cell parameters are only used when they are defined by crystal symmetry. An approximate (isotropic) treatment of cell esds is used for estimating esds involving l.s. planes.
Refinement. Refinement of F^2^ against ALL reflections. The weighted R-factor wR and goodness of fit S are based on F^2^, conventional R-factors R are based on F, with F set to zero for negative F^2^. The threshold expression of F^2^ > 2sigma(F^2^) is used only for calculating R-factors(gt) etc. and is not relevant to the choice of reflections for refinement. R-factors based on F^2^ are statistically about twice as large as those based on F, and R- factors based on ALL data will be even larger.

### Fractional atomic coordinates and isotropic or equivalent isotropic displacement parameters (Å^2^)

**Table:**

	*x*	*y*	*z*	*U*_iso_*/*U*_eq_	
Cl1	0.70519 (2)	0.26502 (10)	0.38081 (9)	0.03119 (16)	
O1	0.41921 (9)	0.7270 (4)	0.6125 (3)	0.0418 (5)	
H1O	0.4080 (14)	0.855 (5)	0.638 (4)	0.041 (10)*	
O2	0.65704 (9)	0.1268 (4)	0.4283 (3)	0.0543 (6)	
O3	0.72121 (12)	0.1930 (5)	0.2130 (3)	0.0615 (7)	
O4	0.69370 (9)	0.5318 (3)	0.3859 (3)	0.0490 (5)	
O5	0.74825 (10)	0.2072 (4)	0.4975 (4)	0.0633 (7)	
N1	0.64637 (12)	0.7547 (5)	0.7092 (4)	0.0383 (6)	
H1N	0.6637 (19)	0.763 (7)	0.624 (7)	0.067 (14)*	
H2N	0.6575 (16)	0.911 (8)	0.768 (6)	0.082 (13)*	
H3N	0.6580 (18)	0.587 (9)	0.759 (6)	0.102 (16)*	
C1	0.47480 (12)	0.7447 (5)	0.6368 (3)	0.0303 (6)	
C2	0.50729 (12)	0.5551 (5)	0.5650 (4)	0.0323 (6)	
H2	0.4911	0.4256	0.5014	0.039*	
C3	0.56328 (12)	0.5591 (5)	0.5878 (4)	0.0347 (6)	
H3	0.5852	0.4326	0.5404	0.042*	
C4	0.58649 (11)	0.7533 (4)	0.6821 (3)	0.0308 (6)	
C5	0.55500 (11)	0.9431 (5)	0.7512 (4)	0.0320 (6)	
H5	0.5714	1.0744	0.8124	0.038*	
C6	0.49832 (11)	0.9382 (5)	0.7292 (4)	0.0324 (6)	
H6	0.4765	1.0651	0.7768	0.039*	
O1W	0.68473 (10)	0.2523 (4)	0.8370 (3)	0.0461 (6)	
H1AW	0.680 (3)	0.245 (11)	0.937 (6)	0.19 (4)*	
H1BW	0.7155 (18)	0.270 (10)	0.793 (8)	0.13 (2)*	
O2W	0.62796 (12)	0.7872 (5)	0.0985 (4)	0.0514 (6)	
H2AW	0.6577 (16)	0.773 (8)	0.172 (5)	0.079 (15)*	
H2BW	0.6131 (18)	0.658 (6)	0.103 (6)	0.070 (14)*	

### Atomic displacement parameters (Å^2^)

**Table:**

	*U*^11^	*U*^22^	*U*^33^	*U*^12^	*U*^13^	*U*^23^
Cl1	0.0311 (3)	0.0332 (3)	0.0292 (3)	0.0050 (2)	0.0005 (3)	−0.0030 (3)
O1	0.0300 (11)	0.0394 (11)	0.0560 (14)	0.0019 (10)	−0.0011 (10)	−0.0022 (11)
O2	0.0455 (12)	0.0505 (11)	0.0667 (15)	−0.0075 (10)	0.0107 (11)	0.0084 (11)
O3	0.0783 (17)	0.0697 (15)	0.0365 (14)	0.0053 (14)	0.0176 (13)	−0.0112 (11)
O4	0.0573 (13)	0.0358 (9)	0.0541 (12)	0.0082 (9)	−0.0003 (13)	0.0001 (11)
O5	0.0484 (15)	0.0774 (16)	0.0640 (17)	0.0242 (13)	−0.0244 (13)	−0.0038 (13)
N1	0.0317 (14)	0.0414 (15)	0.0419 (17)	0.0010 (12)	0.0015 (12)	0.0017 (13)
C1	0.0319 (14)	0.0327 (13)	0.0262 (14)	−0.0010 (12)	−0.0009 (12)	0.0064 (11)
C2	0.0337 (15)	0.0293 (13)	0.0341 (14)	−0.0031 (12)	−0.0005 (12)	−0.0067 (11)
C3	0.0357 (16)	0.0277 (12)	0.0405 (16)	0.0041 (11)	0.0070 (13)	−0.0073 (11)
C4	0.0292 (13)	0.0301 (13)	0.0331 (14)	0.0011 (12)	0.0000 (11)	0.0044 (11)
C5	0.0383 (15)	0.0287 (11)	0.0290 (13)	−0.0009 (11)	−0.0045 (12)	−0.0015 (11)
C6	0.0358 (14)	0.0295 (13)	0.0320 (15)	0.0087 (11)	−0.0014 (11)	−0.0040 (12)
O1W	0.0394 (12)	0.0513 (12)	0.0477 (18)	−0.0040 (12)	−0.0053 (10)	0.0005 (11)
O2W	0.0401 (15)	0.0486 (14)	0.0654 (17)	0.0015 (12)	−0.0133 (12)	0.0038 (13)

### Geometric parameters (Å, °)

**Table:**

Cl1—O3	1.421 (2)	C2—C3	1.375 (4)
Cl1—O5	1.423 (2)	C2—H2	0.9300
Cl1—O2	1.428 (2)	C3—C4	1.379 (4)
Cl1—O4	1.4297 (18)	C3—H3	0.9300
O1—C1	1.370 (3)	C4—C5	1.369 (4)
O1—H1O	0.75 (3)	C5—C6	1.391 (4)
N1—C4	1.473 (4)	C5—H5	0.9300
N1—H1N	0.79 (5)	C6—H6	0.9300
N1—H2N	0.98 (4)	O1W—H1AW	0.79 (5)
N1—H3N	1.00 (5)	O1W—H1BW	0.83 (4)
C1—C6	1.372 (4)	O2W—H2AW	0.93 (4)
C1—C2	1.391 (4)	O2W—H2BW	0.77 (3)
			
O3—Cl1—O5	109.51 (18)	C3—C2—H2	120.0
O3—Cl1—O2	109.27 (17)	C1—C2—H2	120.0
O5—Cl1—O2	109.22 (16)	C2—C3—C4	119.1 (2)
O3—Cl1—O4	109.90 (17)	C2—C3—H3	120.4
O5—Cl1—O4	109.61 (14)	C4—C3—H3	120.4
O2—Cl1—O4	109.31 (13)	C5—C4—C3	121.4 (2)
C1—O1—H1O	105 (3)	C5—C4—N1	119.6 (2)
C4—N1—H1N	114 (3)	C3—C4—N1	119.1 (2)
C4—N1—H2N	110 (2)	C4—C5—C6	119.6 (2)
H1N—N1—H2N	102 (4)	C4—C5—H5	120.2
C4—N1—H3N	109 (3)	C6—C5—H5	120.2
H1N—N1—H3N	103 (4)	C1—C6—C5	119.5 (2)
H2N—N1—H3N	119 (4)	C1—C6—H6	120.2
O1—C1—C6	122.4 (3)	C5—C6—H6	120.2
O1—C1—C2	117.2 (2)	H1AW—O1W—H1BW	124 (7)
C6—C1—C2	120.4 (3)	H2AW—O2W—H2BW	106 (4)
C3—C2—C1	120.0 (3)		
			
O1—C1—C2—C3	−178.6 (2)	C3—C4—C5—C6	1.3 (4)
C6—C1—C2—C3	0.7 (4)	N1—C4—C5—C6	−178.4 (3)
C1—C2—C3—C4	−0.2 (4)	O1—C1—C6—C5	179.1 (2)
C2—C3—C4—C5	−0.8 (4)	C2—C1—C6—C5	−0.2 (4)
C2—C3—C4—N1	178.9 (3)	C4—C5—C6—C1	−0.8 (4)

### Hydrogen-bond geometry (Å, °)

**Table:**

Cg1 is the centroid of the C1–C6 benzene ring.

**Table:**

*D*—H···*A*	*D*—H	H···*A*	*D*···*A*	*D*—H···*A*
O1—H1O···O2W^i^	0.75 (3)	2.10 (3)	2.801 (3)	156 (4)
N1—H1N···O4	0.79 (5)	2.34 (5)	3.016 (4)	144 (4)
N1—H2N···O1W^ii^	0.98 (4)	1.98 (5)	2.951 (4)	168 (4)
N1—H3N···O1W	1.00 (5)	1.97 (5)	2.972 (4)	175 (4)
O1W—H1AW···O3^iii^	0.79 (5)	2.40 (7)	3.089 (3)	146 (8)
O1W—H1BW···O5	0.83 (4)	2.47 (6)	3.083 (4)	132 (5)
O2W—H2AW···O4	0.93 (4)	2.28 (4)	3.068 (4)	143 (4)
O2W—H2BW···O1^iv^	0.77 (3)	2.17 (3)	2.937 (3)	173 (5)
C2—H2···Cg1^iv^	0.93	2.88	3.677 (3)	144

Symmetry codes: (i) −*x*+1, −*y*+2, *z*+1/2; (ii) *x*, *y*+1, *z*; (iii) *x*, *y*, *z*+1; (iv) −*x*+1, −*y*+1, *z*−1/2.
